# Complicated acute appendicitis presenting as a rapidly progressive soft tissue infection of the abdominal wall: a case report

**DOI:** 10.1186/s13256-016-1122-1

**Published:** 2016-12-01

**Authors:** Corinne Beerle, Hans Gelpke, Stefan Breitenstein, Ralph F. Staerkle

**Affiliations:** Department of Surgery, Clinic for Visceral and Thoracic Surgery, Kantonsspital Winterthur, Winterthur, Switzerland

**Keywords:** Perforated appendicitis, Abscess of the abdominal wall, Case report, Soft tissue infection

## Abstract

**Background:**

We report a case of a rare complication of acute appendicitis with perforation through the abdominal wall. The case points out that an intraabdominal origin should be considered in patients presenting with rapidly spreading soft tissue infections of the trunk.

**Case presentation:**

A 58-year-old European woman presented to our hospital with a 1-week history of severe abdominal pain accompanied by rapidly spreading erythema and emphysema of the lower abdomen. On admission, the patient was in septic shock with leukocytosis and elevation of C-reactive protein. Among other diagnoses, necrotizing fasciitis was suspected. Computed tomography showed a large soft tissue infection with air-fluid levels spreading through the lower abdominal wall. During the operation, we found a perforated appendicitis breaking through the fascia and causing a rapidly progressive soft tissue infection of the abdominal wall. Appendicitis was the origin of the soft tissue infection. The abdominal wall was only secondarily involved.

**Conclusions:**

Even though perforated appendicitis as an etiology of a rapidly progressive soft tissue infection of the abdominal wall is very rare, it should be considered in the differential diagnosis of abdominal wall cellulitis. The distinction between rapidly spreading subcutaneous infection with abscess formation and early onset of necrotizing fasciitis is often difficult and can be confirmed only by surgical intervention.

## Background

Soft tissue infections are very common in adult patients. Usually, they present with redness, swelling, and pain [1]. The clinical distinction between simple cellulitis and a rapidly spreading soft tissue infection, sometimes caused by gas-forming organisms and evolving into a life-threatening necrotizing fasciitis, is often difficult [2]. Laboratory tests may not differentiate the two diagnoses, but performing imaging using computed tomography (CT) or using magnetic resonance imaging (MRI) with extensive stranding of the subcutaneous tissue, gas, and the rapidly developing clinical picture of systemic inflammatory response may help to obtain the correct diagnosis [1, 3].

Acute appendicitis is one of the most common surgical diseases and is usually managed by appendectomy with low morbidity and mortality. Complications such as perforation with abscess formation and localized or four-quadrant peritonitis occur in about 15% of patients [4, 5]. However, the perforation of an appendicular abscess through the abdominal wall and an ensuing soft tissue infection are rare events nowadays.

This case report describes a rare complication of an appendicular abscess perforating into the abdominal wall and its surgical management. The case demonstrates that an intraabdominal origin has to be considered in the differential diagnosis of soft tissue infection of the abdominal wall.

## Case presentation

A 58-year-old European woman with a medical history of hypertension, asthma, and depression presented to our emergency department with abdominal pain of 1 week’s duration. Over the preceding 3 days, the patient’s condition had declined rapidly with general weakness and severe escalating but rather superficial abdominal pain.

On admission, the patient complained of abdominal pain. Her temperature was 35.1°C, blood pressure was 63/46 mmHg, and heart rate was 88 beats/minute. She was not oriented to time and space. Her physical examination revealed truncal obesity (36.3 kg/m^2^) with an irregular patch of erythema and tenderness in the right lower quadrant, and crepitation on palpation. The skin in the area of the erythema was hypoesthesic.

**Fig. 1:**
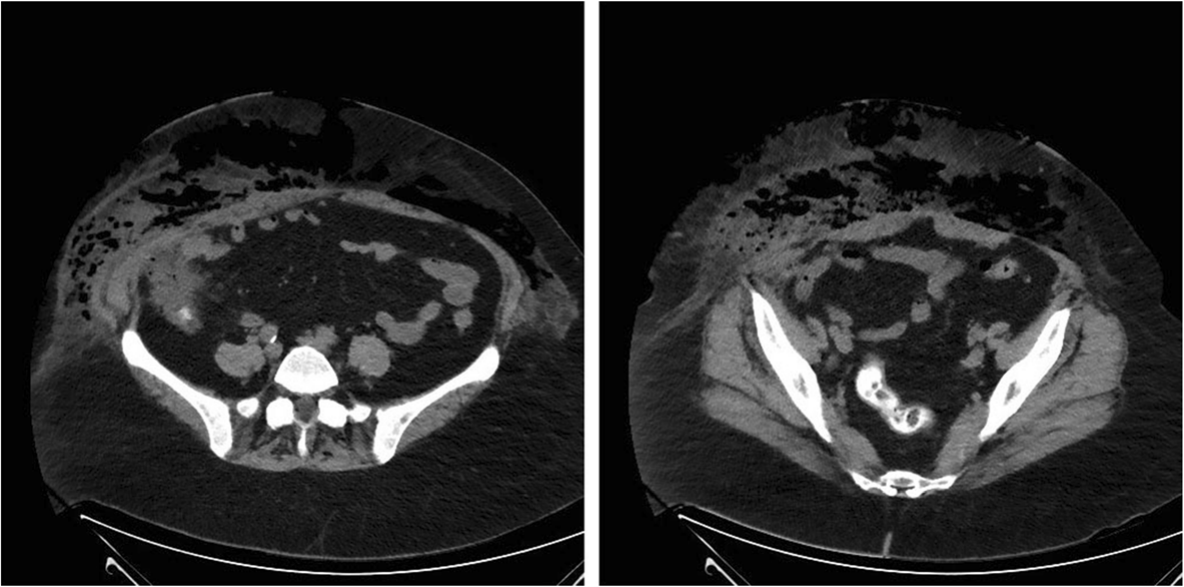
Computed tomography performed on the patient’s admission shows extensive emphysema in the subcutaneous tissue without any signs of an intraabdominal infection

Initial laboratory test results showed a C-reactive protein of 439 mg/L as well as a white blood cell count of 50 g/L with 48% neutrophils. Her serum creatinine was 239 μmol/L, sodium was 131 mmol/L, and potassium was 3.3 mmol/L. An immediate abdominal CT was performed. No venous contrast was used, owing to the patient’s increased creatinine and oliguria. CT showed extensive subcutaneous emphysema in the right and left lower quadrants with small pockets of air-fluid levels (Fig. 1). There was no intraabdominal free fluid, free air, or any sign of intraabdominal inflammation. However, a contiguity of the cecum to the abdominal wall and to the subcutaneous collection was noted.

**Fig. 2:**
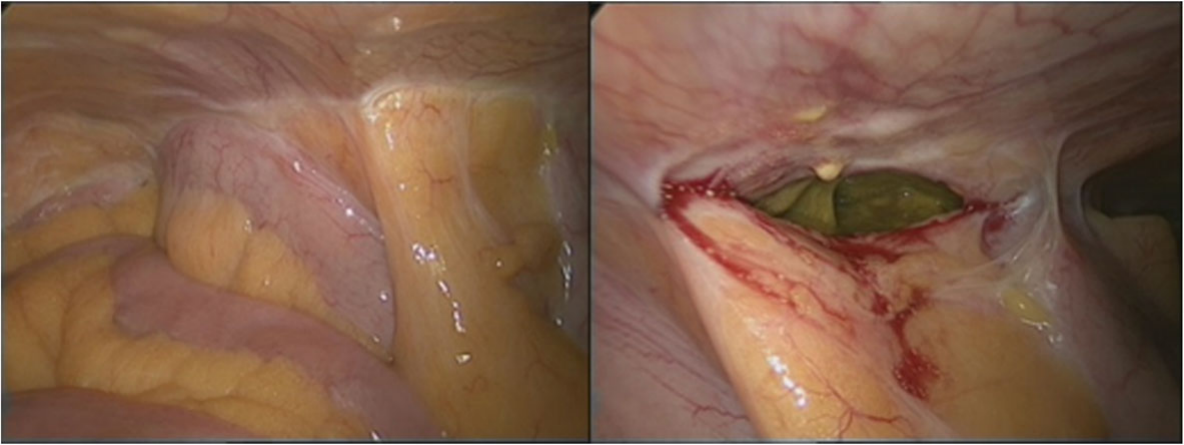
Diagnostic laparoscopy without any signs of intraabdominal infection and ileocecal area adherent to the abdominal wall

**Fig. 3:**
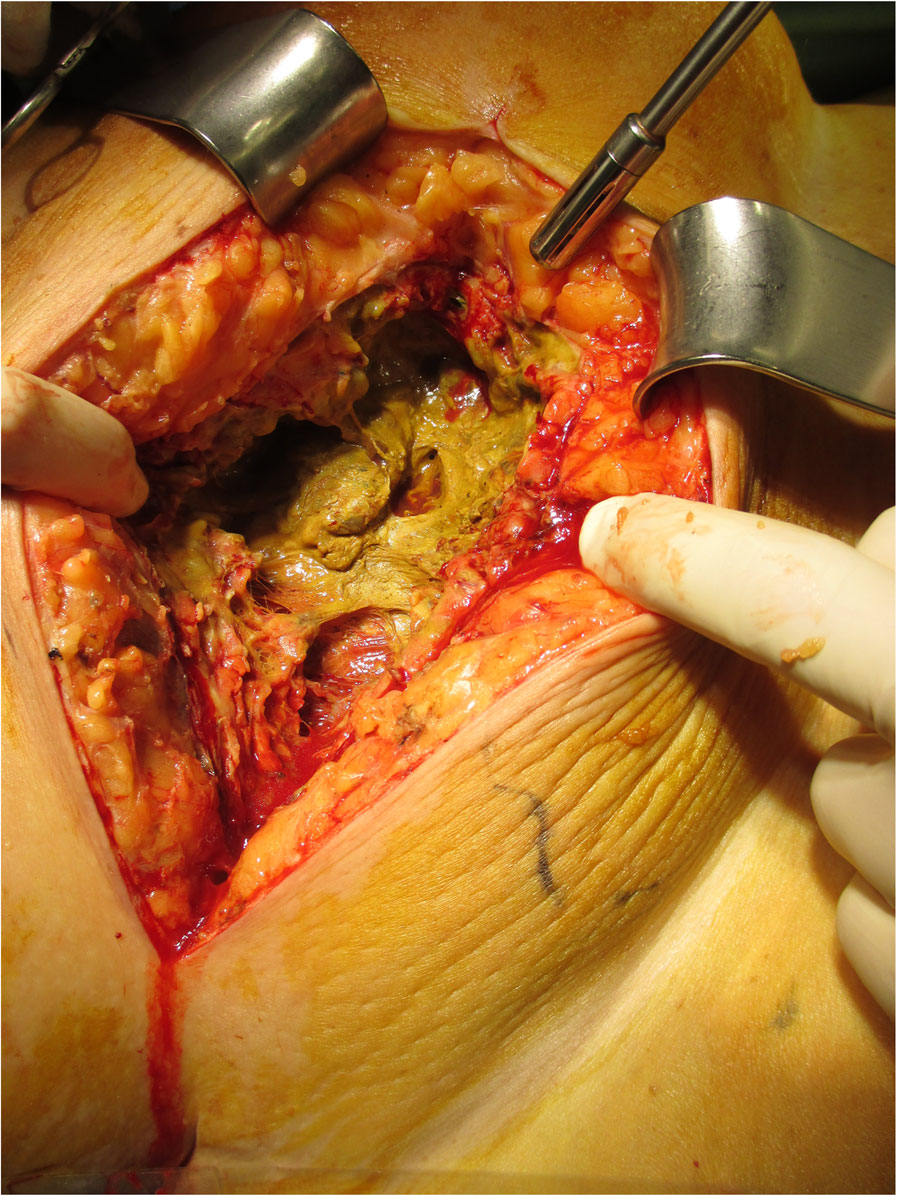
Stool in the subcutaneous tissue from the perforated appendix

**Fig. 4:**
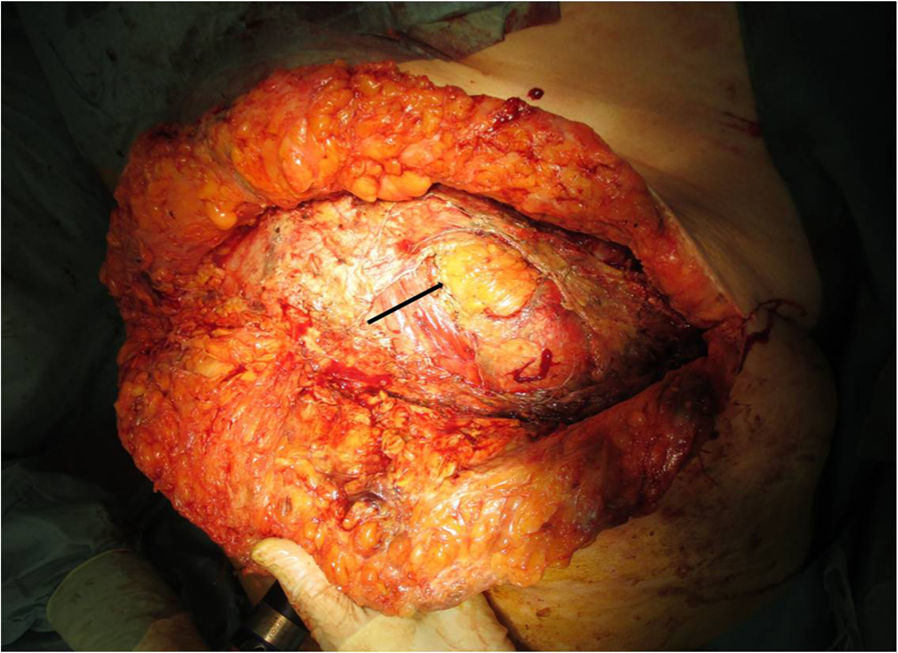
Situs after radical debridement of the subcutaneous tissue. However, the fascia is not involved. The arrow marks the site of the perforation through the abdominal wall

The differential diagnosis included necrotizing fasciitis, a tumor of the appendix or the cecum with perforation into the abdominal wall, or acute perforated appendicitis. After stabilization of the patient’s septic shock with 3 L of normal saline, she was urgently taken to the operating theater. First, diagnostic laparoscopy using a midline supraumbilical open approach was performed to evaluate for a potentially intraabdominal origin of the abdominal wall infection. It revealed an ileocecal area adherent to the abdominal wall without any signs of intraabdominal infection (Fig. 2). However, after lateral mobilization of the cecum, an abscess cavity with perforation into the abdominal wall was found (Fig. 3). After horizontal incision and debridement of the subcutaneous tissue were performed, the defect in the muscular layers of the abdominal wall measured 7 × 7 cm (Fig. 4). Perforated appendicitis appeared to be the source of the infectious process. The stump of the appendix was closed by a suture ligation. At that point, an ileocolic resection with a diverted ileostomy was not considered necessary, owing to the lack of intraabdominal infection and the fact that it would have been complicated by the extensive defect and inflammation of the abdominal wall as well as by the obesity of the patient. The defect of the abdominal wall was covered using an omental patch. A dressing with Betadine (Purdue Products, Stamford, CT, USA)-soaked gauze was applied to the debrided wound from the outside.

**Fig. 5:**
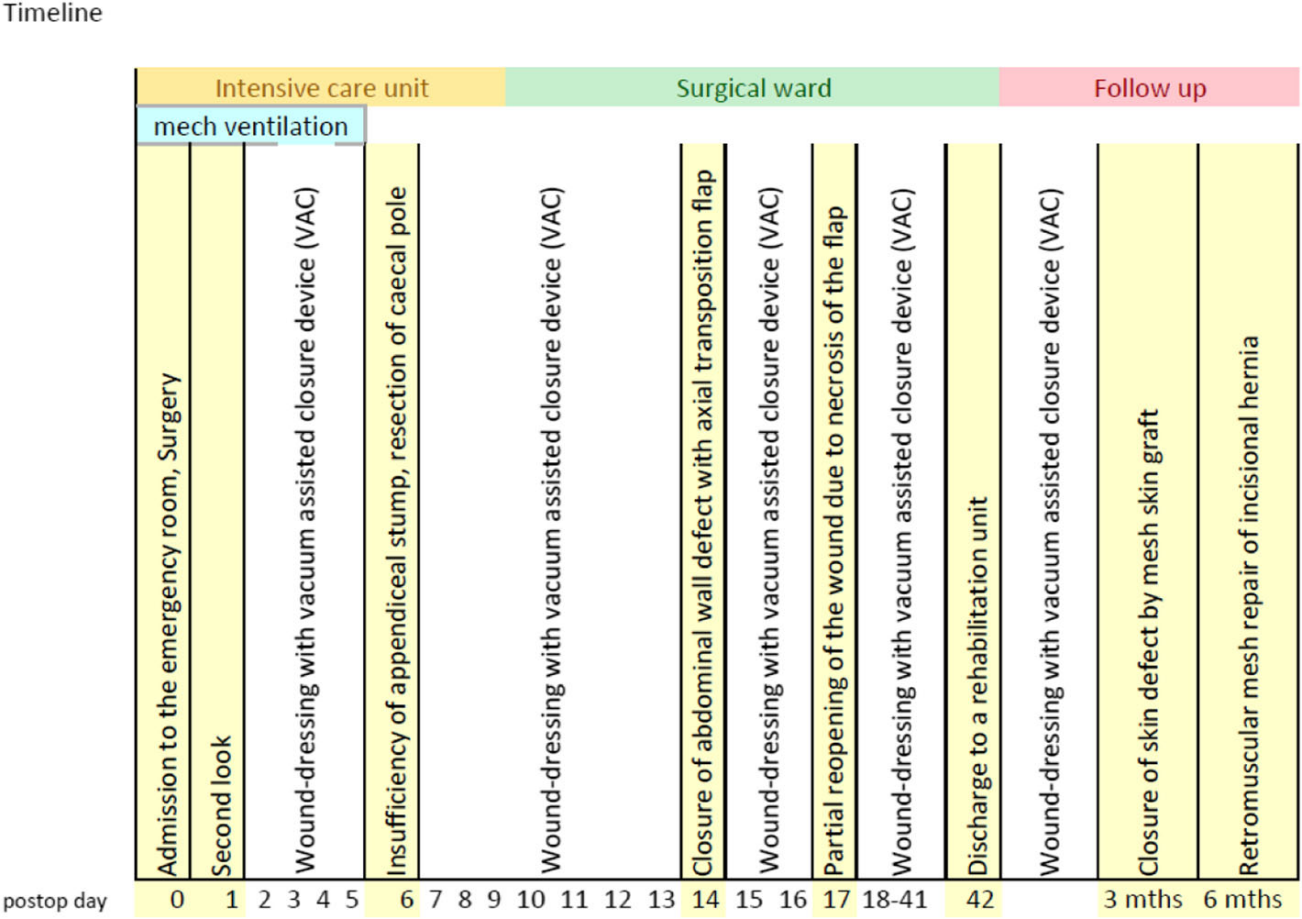
Timeline

Postoperatively, the patient was admitted to the intensive care unit in continuing septic shock with multiorgan dysfunction, including acute kidney and liver failure as well as septic encephalopathy. She remained on mechanical ventilation for 5 days. Bacteriological results from blood cultures as well as abscess and peritoneal fluids were positive for *Escherichia coli*. Intravenous antibiotic therapy with piperacillin/tazobactam 4.5 g three times daily was started immediately after surgery and continued for 20 days. After 24 h, a second surgical look of the abdominal wall was performed in the operating theater with additional debridement of necrotic subcutaneous tissue. Afterward, the wound was dressed with a vacuum-assisted closure (VAC) device (V.A.C.® GranuFoam^TM^; Acelity, San Antonio, TX, USA) according to standard procedure in our clinic. On the sixth postoperative day (POD), the patient was taken to the operating theater for a change of the VAC dressing. Unfortunately, we discovered a leakage of feces through the abdominal defect caused by an insufficiency of the appendicular stump. This led us to decide to perform a cecal wedge resection because the cecum showed good blood circulation and no signs of infection. According to our opinion, an ileostomy was not necessary in this situation. Performing an ileostomy clearly would have been very demanding in this severely obese patient with a huge defect of the abdominal wall.

Because of the highly contaminated wound, we used a biological mesh (STRATTICE™ Reconstructive Tissue Matrix; LifeCell/Acelity, Branchburg, NJ, USA) to cover the defect with no further reconstruction of the abdominal wall. The cutaneous and subcutaneous defect was closed by an axial transposition flap (skin and subcutaneous tissue) from the right upper abdomen on POD 14.

Three days later, the wound had to be partially reopened because of necrosis of the transposition flap. Again, a wound dressing with a VAC device was applied. The dressing was changed regularly, first with the patient under general anesthesia in the operating theater, later at bedside.

The patient was transferred to the surgical ward on POD 10 and discharged on POD 42 to a rehabilitation unit in improved general condition (Fig. 5)﻿. The further changes of the VAC dressing were performed on an outpatient basis. Three months after primary admission, the skin defect was closed by mesh skin graft.

Six months after the closure of the abdominal wall defect with a biological mesh, the patient developed an incisional hernia. A retromuscular hernia repair was performed with a mesh that is manufactured from approximately equal parts of absorbable poliglecaprone 25 monofilament fiber and nonabsorbable polypropylene monofilament fiber (ULTRAPRO; Ethicon/Johnson & Johnson, Somerville, NJ, USA) because it is the standard mesh for incisional hernias in our hospital. The patient’s postoperative course was uneventful, and she fully recovered.

## Discussion

Appendicitis is one of the most common causes of abdominal pain and can affect all age groups. Perforation is seen in 20–30% of these patients. Once perforation has occurred, complications such as wound infection, abscess formation, and peritonitis are frequent [6, 7]. An abscess of the abdominal wall due to perforated appendicitis represents a rare but dangerous complication with increased mortality [8]. Early open or laparoscopic removal of the inflamed appendix is the most effective method of preventing complications [4, 6, 8]. In retrospect, performing an ileocolic resection with diverting ileostomy instead of only removal of the inflamed appendix would have spared our patient the complication of an appendicular stump insufficiency.

To the best of our knowledge, only two similar cases with gas-forming abscesses of the abdominal wall due to perforated appendicitis have been published [8, 9]. Authors of other publications reported cases of acute appendicitis complicated by necrotizing fasciitis of the abdominal wall or of the lower limb, including one case of Fournier’s gangrene [8].

The distinction between rapidly spreading soft tissue infection and the early onset of necrotizing fasciitis remains challenging. While rapid and extensive surgical treatment of fasciitis can be lifesaving, soft tissue infection without abscess formation requires primarily antibiotic treatment [2].

Soft tissue infections are very common in primary care [1]. They generally involve skin and subcutaneous tissue and most commonly occur in the lower extremities [1]. Patients present with pain, redness, and swelling of the involved skin. Fever and general symptoms are most frequently mild [1].

In contrast, necrotizing soft tissue infections may spread rapidly and develop along fascial planes, occasionally sparing skin and underlying muscle [1, 3, 10]. These patients experience pain, fever, rapid deterioration, gas or crepitus, and a systemic inflammatory response syndrome with elevated inflammatory markers [1, 11, 12].

Our patient showed rapidly progressing erythema with severe pain, hemodynamic instability, oliguria, crepitation, and septic shock syndrome, which were highly suspicious for necrotizing fasciitis. A Risk Indicator for Necrotizing Fasciitis (LRINEC) score developed to differentiate between soft tissue infection and necrotizing fasciitis was 8 points, representing a risk of over 90% for necrotizing fasciitis. This score includes routine laboratory values, such as C-reactive protein, white blood cell count, sodium, creatinine, glucose, and hemoglobin levels [2, 10].

The use of CT is helpful to confirm the diagnosis of a soft tissue infection, especially when air associated with fluid collection is found. Additionally, it can provide information about involvement of intraabdominal organs as a source for the infection [13–15]. MRI has been shown to be the most precise imaging method to differentiate between a simple subcutaneous infection and necrotizing fasciitis [1, 3]. However, MRI examination is time-consuming and not always available. Nevertheless, as shown in our patient’s case, final confirmation or exclusion of the diagnosis of necrotizing fasciitis can be achieved only during surgical exploration with histological and microbiological workup [1, 3].

## Conclusions

Even though perforated appendicitis as an etiology of a spreading subcutaneous infection of the abdominal wall is a rare entity, intraabdominal causes (e.g., appendicitis, diverticulitis) ought to be considered when faced with a patient with abdominal wall cellulitis, and cross-sectional abdominal imaging should be performed prior to surgical debridement.

## Abbreviations

CT: Computed tomography
LRINEC: Laboratory Risk Indicator for Necrotizing Fasciitis
MRI: Magnetic resonance imaging
POD: Postoperative day
VAC: Vacuum-assisted closure

## References

[1] Gunderson CG. Cellulitis: definition, etiology, and clinical features. Am J Med. 2011;124(12):1113–22. doi:10.1016/j.amjmed.2011.06.028. 22014791

[2] Wong CH, Khin LW, Heng KS, Tan KC, Low CO. The LRINEC (Laboratory Risk Indicator for Necrotizing Fasciitis) score: a tool for distinguishing necrotizing fasciitis from other soft tissue infections. Crit Care Med. 2004;32(7):1535–41. 10.1097/01.ccm.0000129486.35458.7d 15241098

[3] Schmid MR, Kossmann T, Duewell S. Differentiation of necrotizing fasciitis and cellulitis using MR imaging. AJR Am J Roentgenol. 1998;170(3):615–20. doi:10.2214/ajr.170.3.9490940. 9490940

[4] Simpson J, Samaraweera AP, Sara RK, Lobo DN. Acute appendicitis – a benign disease? Ann R Coll Surg Engl. 2008;90(4):313–6. doi:10.1308/003588408X285973. PMC2647194 18492396

[5] Drake FT, Mottey NE, Farrokhi ET, Florence MG, Johnson MG, Mock C, *et al*. Time to appendectomy and risk of perforation in acute appendicitis. JAMA Surg. 2014;149(8):837–44. doi:10.1001/jamasurg.2014.77. PMC4160117 24990687

[6] Bat O, Kaya H, Celik HK, Sahbaz NA. Clinical results of laparoscopic appendectomy in patients with complicated and uncomplicated appendicitis. Int J Clin Exp Med. 2014;7(10):3478–81. PMC4238537 25419386

[7] Mukoyama S, Mukai M, Yasuda S, Oida Y, Himeno S, Nishi T, *et al*. A successfully treated case of severe necrotizing fasciitis caused by acute appendicitis: a case report. Tokai J Exp Clin Med. 2003;28(3):139–43. 15055407

[8] Fanning DM, Barry M, O’Brien GC, Leahy AL. Perforation of a retrocaecal appendix presenting clinically as a right lumbar abscess. Surgeon. 2007;5(6):368–70. 10.1016/s1479-666x(07)80090-4 18080612

[9] Ishigami K, Khanna G, Samuel I, Dahmoush L, Sato Y. Gas-forming abdominal wall abscess: unusual manifestation of perforated retroperitoneal appendicitis extending through the superior lumbar triangle. Emerg Radiol. 2004;10(4):207–9. doi:10.1007/s10140-003-0314-3. 15290493

[10] Gujral S, Hughes JM, Wiberg A. Necrotizing fasciitis. Eplasty. 2014;14:ic15. PMC4034121 24917900

[11] Green RJ, Dafoe DC, Raffin TA. Necrotizing fasciitis. Chest. 1996;110(1):219–29. 10.1378/chest.110.1.219 8681631

[12] McHenry CR, Piotrowski JJ, Petrinic D, Malangoni MA. Determinants of mortality for necrotizing soft-tissue infections. Ann Surg. 1995;221(5):558–65. 10.1097/00000658-199505000-00013 PMC1234638 7748037

[13] Taif S, Alrawi A. Missed acute appendicitis presenting as necrotising fasciitis of the thigh. BMJ Case Rep. 2014;2014:bcr2014204247. doi:10.1136/bcr-2014-204247. PMC4025401 24792028

[14] Marron CD, Khadim M, McKay D, Mackle EJ, Peyton JW. Amyand’s hernia causing necrotising fasciitis of the anterior abdominal wall. Hernia. 2005;9(4):381–3. doi:10.1007/s10029-005-0320-3. 15703857

[15] Takeda M, Higashi Y, Shoji T, Hiraide T, Maruo H. Necrotizing fasciitis caused by a primary appendicocutaneous fistula. Surg Today. 2012;42(8):781–4. 10.1007/s00595-012-0140-x PMC3396344 22318638

